# Estimation of soybean yield parameters under lodging conditions using RGB information from unmanned aerial vehicles

**DOI:** 10.3389/fpls.2022.1012293

**Published:** 2022-12-13

**Authors:** Dong Bai, Delin Li, Chaosen Zhao, Zixu Wang, Mingchao Shao, Bingfu Guo, Yadong Liu, Qi Wang, Jindong Li, Shiyu Guo, Ruizhen Wang, Ying-hui Li, Li-juan Qiu, Xiuliang Jin

**Affiliations:** ^1^ The National Key Facility for Crop Gene Resources and Genetic Improvement (NFCRI)/Key Laboratory of Crop Gene Resource and Germplasm Enhancement (MOA)/Key Laboratory of Soybean Biology (Beijing) (MOA), Institute of Crop Science, Chinese Academy of Agricultural Sciences, Beijing, China; ^2^ Nanchang Branch of National Center of Oil Crops Improvement, Jiangxi Province Key Laboratory of Oil Crops Biology, Crops Research Institute of Jiangxi Academy of Agricultural Sciences, Nanchang, China; ^3^ College of Agriculture, Northeast Agricultural University, Harbin, China; ^4^ National Nanfan Research Institute (Sanya), Chinese Academy of Agricultural Sciences, Sanya, China

**Keywords:** UAV, yield, soybean, lodging, machine learning

## Abstract

The estimation of yield parameters based on early data is helpful for agricultural policymakers and food security. Developments in unmanned aerial vehicle (UAV) platforms and sensor technology help to estimate yields efficiency. Previous studies have been based on less cultivars (<10) and ideal experimental environments, it is not available in practical production. Therefore, the objective of this study was to estimate the yield parameters of soybean (Glycine max (L.) Merr.) under lodging conditions using RGB information. In this study, 17 time point data throughout the soybean growing season in Nanchang, Jiangxi Province, China, were collected, and the vegetation index, texture information, canopy cover, and crop height were obtained by UAV-image processing. After that, partial least squares regression (PLSR), logistic regression (Logistic), random forest regression (RFR), support vector machine regression (SVM), and deep learning neural network (DNN) were used to estimate the yield parameters. The results can be summarized as follows: (1) The most suitable time point to estimate the yield was flowering stage (48 days), which was when most of the soybean cultivars flowered. (2) The multiple data fusion improved the accuracy of estimating the yield parameters, and the texture information has a high potential to contribute to the estimation of yields, and (3) The DNN model showed the best accuracy of training (R^2^=0.66 rRMSE=32.62%) and validation (R^2^=0.50, rRMSE=43.71%) datasets. In conclusion, these results provide insights into both best estimate period selection and early yield estimation under lodging condition when using remote sensing.

## 1 Introduction

Global soybean (*Glycine max* (L.) Merr.) production steadily increased during the last two decades, primarily in the United States, Brazil, Argentina, China, Paraguay, and India, which accounted for 94.3% of the global soybean production in 2021 (Agriculture, 2021). Accurately estimating the yields at the early crop growth stage is important for the government to deploy the appropriate share of imports.

Traditional ways of estimating soybean yield rely on destructive sampling and manual experience (Jin et al., 2021), they are time-consuming. Traditional methods of estimating yields utilize plot yield as a qualitative indices (Geipel et al., 2014), it is strongly influenced by the environment and a variety of biotic factors (Hamblin et al., 1978) if there are a lot of cultivars and a small plot area. So more stable yield parameters (grain number of seeds per plant and grain weight per plant) were used as field yield study data (Hamblin et al., 1978). Most types of yield estimation neglect the effects of micro-environmental factors owing to the large plot areas and a limited number of cultivars (Ji et al., 2022), so they were highly accurate in approximating the mean value of the yield of cultivars but not were not effective at truly identifying germplasm resources.

In recent years, high-throughput phenotyping has garnered increasing attention, particularly the use of unmanned aerial vehicles (UAVs) as a phenotyping platform combined with high-quality image sensors (Guo et al., 2021). High-throughput UAV phenotyping can not only reduce the threshold of traditional phenotyping but can also efficiently locate genes (Sukumaran et al., 2018) and provide basic data support for molecular biology and the genetic breeding of crops (Venkatalaxmi et al., 2004). With the developments of multi-spectral sensors (Maimaitijiang et al., 2017), hyperspectral sensors (Yue et al., 2018) and lidar (Jin et al., 2015), it can provide tens of millions of MB data support for the identification of diverse phenotypes and genotypes and molecular breeding (Potgieter et al., 2017). But previous yield estimates have utilized relatively inaccessible multispectral, hyperspectral (Paulus and Mahlein, 2020), and even radar data, which are highly accurate but unsuitable in real field plots.

In previous studies the UAV-derived datasets were used to estimate multiple vegetation traits (Maimaitijiang et al., 2017) and yield. The primary methods of estimating yields include physical models and machine learning models. Crop growth models have been proposed to estimate crop yields under different scenarios, including climate, genotype, soil properties, and management factors (Araus et al., 2021). For example, Ma et al. (2022) used the single algorithm for yield (SAFY) crop growth model to estimate the yields of wheat (*Triticum aestivum* L.). The results obtained an R^2^ of 0.73, 0.83, and 0.49 for the leaf area index (LAI), biomass, and yield with root mean square error (RMSE) values of 0.72, 1.13 t/ha and 1.14 t/ha (Ma et al., 2022). Jin et al. (2022) conducted research on the ChinaAgrosys crop model on wheat. The R^2^ of estimated maturity, LAI, and yield were higher than 0.73, 0.44, and 0.60 (Jin et al., 2022). These models can provide reasonable explanations for a variety of biochemical mechanisms and responses, but there are deficiencies in the input parameters to estimate the yield under complex and unpredictable scenarios (Zhao et al., 2013). In addition, previous studies estimated yields using multispectral images for machine learning or deep learning. Khaki and Wang (2019) applied deep neural networks to estimate the yield of maize (*Zea mays* L.) hybrids using environmental data. The use of weather data reduced the RMSE to 11% of the average yield and 46% of the standard deviation (SD) (Khaki and Wang, 2019). Feng et al. (2021) proposed a geographically and temporally weighted neural network (GTWNN) model for 12 years of data from 2008 to 2019, and the GTWNN outperformed other models (Feng et al., 2021). Maimaitijiang et al. (2020) used five machine learning algorithms to estimate the yield of soybeans, and DNN-F2 was the most accurate with an R^2^ of 0.72 and a relative root mean square error (RMSE%) of 15.90% (Maimaitijiang et al., 2020). PLSR and RFR were used for quantifying soil salinity (Wang et al., 2018). The validation accuracy showed that the RFR model performed better than the model of PLSR. The most effective model was built based on the 1.5th order derivative of RF with respect to absorbance with the best values of R^2^ (0.93), RMSE (4.57 dS m(-1)). SVM is mostly used for disease classification and monitoring, and known studies include A. O. Conrad et al. (2020) used SVM to build and evaluate the accuracy of disease prediction models based on supervised classification. Sparse partial least squares discriminant analysis was used to confirm the results. The most accurate model comparing mock-inoculated and inoculated plants was SVM-based with an overall test accuracy of 86.1% (N = 72), while the most accurate SVM model had an overall test accuracy of 73.3% (N = 105) (Conrad et al., 2020).

In summary, although studies on estimating yield have been conducted quite frequently, these data are basically from a limited number of cultivars (<10), and thus, are not widely applicable and can only be considered to be an exploration of the technique. In addition, they rarely consider the yield status under specific environmental conditions, while the constant environmental variability in the field has crucial effects on yield.

Therefore, in this study, the performance of five algorithms to estimate yield parameters under lodging conditions was estimated, and the optimal time point to estimate yield during the early dates was studied based on more than 1,500 soybean germplasm cultivars. The aims of this study were to 1) Find the most suitable time point to estimate the yield parameters of soybean. 2) Test the multi-data fusion of vegetation indices, canopy cover, and crop height based on a Digital Elevation Model (DEM), lodging, and texture indices to estimate yield using different machine learning algorithms, and 3) To assess the effect of lodging on the grain number of seeds per plant and grain weight per plant.

## 2 Materials and methods

### 2.1 Materials

#### 2.1.1 Study area

**Figure 1:**
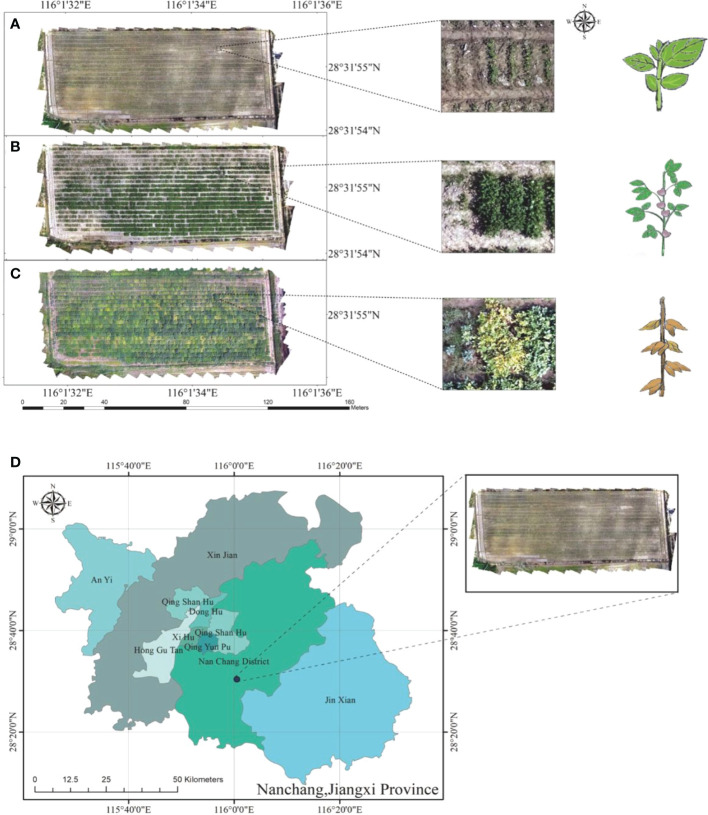
Study area. **(A)** RGB orthomosaic map on August 1, 2020. **(B)** RGB orthomosaic map on August 20, 2020. **(C)** RGB orthomosaic map on September 24, 2020. **(D)** The test field was in Nanchang, Jiangxi Province, China.

The experiment was conducted from July to November 2020 in Nanchang, Jiangxi Province, China (115°27’ ~116°35’ E, 28°10’ ~29° 11’ N), which has a humid subtropical monsoon climate, abundant precipitation of 2,167.9 mm in 2020 (Nanchang weather station NO.58606), average annual sunshine of 1,772~1,845 hours, long summer and winter and short spring and autumn throughout the year, and an average annual temperature of 17.0°C-17.7°C. The experimental terrain is a plain, with an average elevation of 22 m. The experimental area of 0.92 acres was located in the southwestern suburbs of Nanchang City (**Figure 1**).

A total of 1,615 cultivars and 190 plots of controls (30 cultivars) were sown on July 15, 2020, and the UAV photos were collected at 17 time points (Li et al., 2022), which represented different growing stages. Some of those cultivars that had either a low germination or were under abiotic/biotic stresses were also studied to maintain high diversity, instead of dropping in the previous study (Li et al., 2022). The soybean cultivars came from worldwide and the largest number of cultivars were from China (70%), followed by the USA and Europe, and covered a wide range of ecotypes. The growth states of the different cultivars differed significantly. The plots were 1.8 m × 0.8 m with two rows and 10 cm plant spacing, so there were 1,805 plots in total. They were separated by furrows. Conventional N P K compound fertilizer of potassium sulfate 15:15:15 was applied once (K_2_O_4_S, 50%, 112.5kg/ha).

#### 2.1.2 Image collection parameters

A DJI Phantom 4 (DJI Technology Co., Shenzhen, China) was used to collect the image data. As detailed in **Table 1**, the image was outputted in the tiff format. The UAV (unmanned aerial vehicle) platform was implemented with an autopilot system (Şahin et al., 2022) to execute a predefined flight from 10:00 to 14:00 every 3 days for 20 minutes. To avoid the partial loss of image texture feature information owing to cloud cover, weather with stable solar radiation intensity and a clear and cloudless sky was selected to acquire the images. The images had a resolution of 1,600*1,300 pixels, and all the flight missions were planned at the flight altitude and speed of 12 m and 1.2 m/s with the lateral and forward overlaps (Kose and Oktay, 2020) of 75% and 60% (before 13 September)/75% (after 13 September), respectively. Real-time kinematic (RTK) was used for positioning. The RTK accuracy that was enabled was ± 0.1 m vertically and horizontally (Oktay and Coban, 2017). The quality of images was checked after the flight.

**Table 1:** Drone technical details.

Project	Technical details
Time per flight	Approximately 20 minutes
Hover accuracy	When RTK is enabled and RTK is working properly: Vertical: ± 0.1 m; Horizontal: ± 0.1 m
Controlled rotation range	Pitch: -90 degrees to +30 degrees
Image sensor	6 1/2.9-inch CMOS; Single sensor: 2.08 million effective pixels (2.12 million total pixels)
Lens	FOV: 62.7 degrees; Focal length: 5.74 mm (35 mm format equivalent: 40 mm); Infinity fixed focal length; Aperture : f/2.2
The resolution of the image	1600×1300(4:3.25)

CMOS, complementary metal oxide semiconductor (an image sensor); RTK, real-time kinematic.

The experimental field design required that the soybean planting site be treated with weed control in advance. Therefore, the effect of weeds on each indices of the image was not considered in this study. The 0.5 m × 0.5 m image ground control points (GCPs) set between the different routes were kept constant throughout the soybean growing stages. Agisoft PhotoScan software (Agisoft LLC, St. Petersburg, Russia) was used to stitch the UAV digital images. The software can perform image geometry correction and eliminate the effects of UAV attitude changes based on GCPs.

### 2.2 Methods

#### 2.2.1 Overview for data process

**Figure 2:**
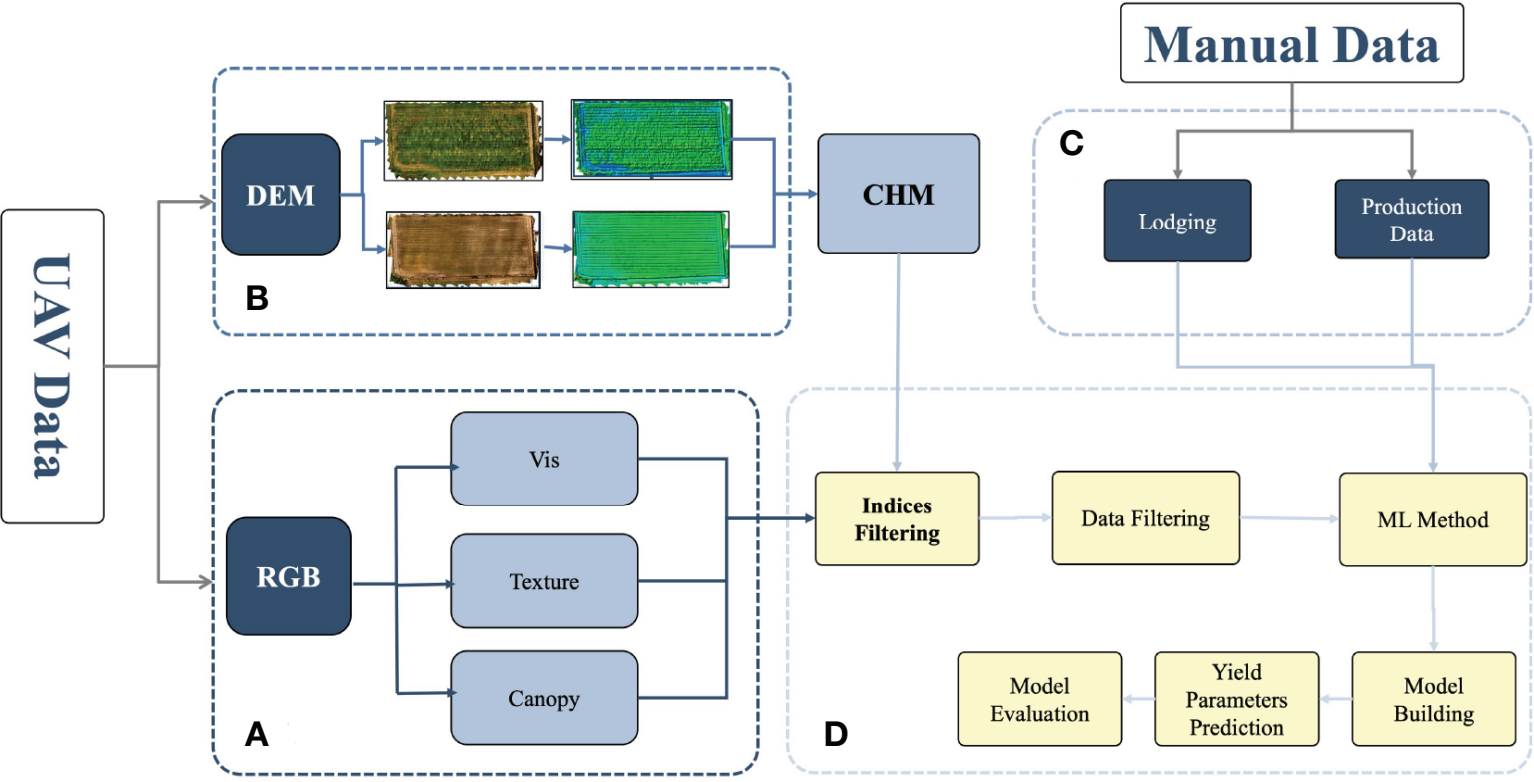
Flowchart for estimating the yield parameters. **(A)** RGB indices extraction process. **(B)** Calculation of the plant height using DEM. **(C)** Manual data, including lodging and yield parameters, such as the grain number of seeds per plant and grain weight per plant. **(D)** Data cleaning and model building. DEM, digital elevation model; RGB, red green blue.

This work used the UAV data and manually collected data. The UAV data consisted of RGB (red, green, blue) and DEM (digital elevation model) data. The degree of lodging and yield parameters (grain number of seeds per plant and grain weight per plant) were collected manually. The RGB data were calculated for vegetation indices, including texture information and canopy cover. The normalized results of crop height were calculated using DEM. After the Indices and data had been screened, the datasets were divided into a training dataset (70%) and a testing dataset (30%), followed by modeling using five machine learning algorithms, and the performance of the models was finally evaluated to estimate the yield parameters. The flowchart is shown in **Figure 2**, and the details are discussed below.

#### 2.2.2 Measurements of yield parameters

**Figure 3:**
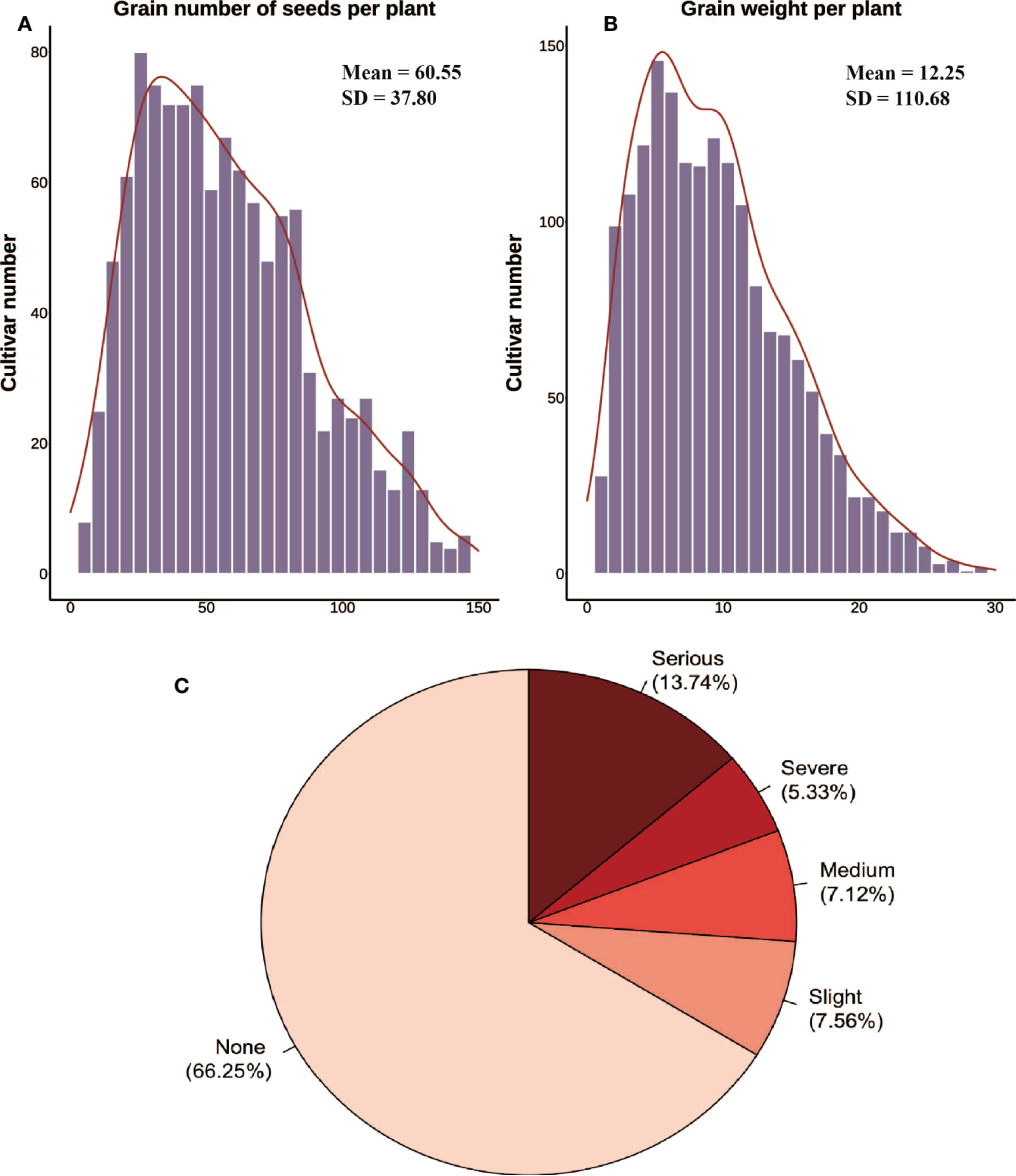
Yield parameters and inversions collected manually. **(A, B)** Histogram distribution of the grain number of seeds per plant and the grain weight per plant from manual surveys. **(C)** Lodging classification and percentage.

The yield parameters used in this work were the grain number of seeds per plant and the grain weight per plant **Figure 3A**. The mean value was calculated after 10~15 plants were randomly selected in each plot after harvest. The lodgings were monitored by experts from the Jiangxi Academy of Agricultural Sciences (Nanchang, China) to ensure reliable data during the whole growth stage. The levels of lodging severity were divided into five classifications (1-5) (Kato et al., 2021). The degree of tilting stalks at maturity was used to divide the plots with level 1 indicating that all the plants in plot were upright, and level 5 indicating that all the plants in plot had lodged **Figures 3B, C**. Stalk strength, root traits, and biological yield, which are closely related to lodging, were not considered mechanistically. Thus, a comprehensive index approach was not utilized to evaluate lodging resistance.

#### 2.2.3 Image pre-processing

**Figure 4:**
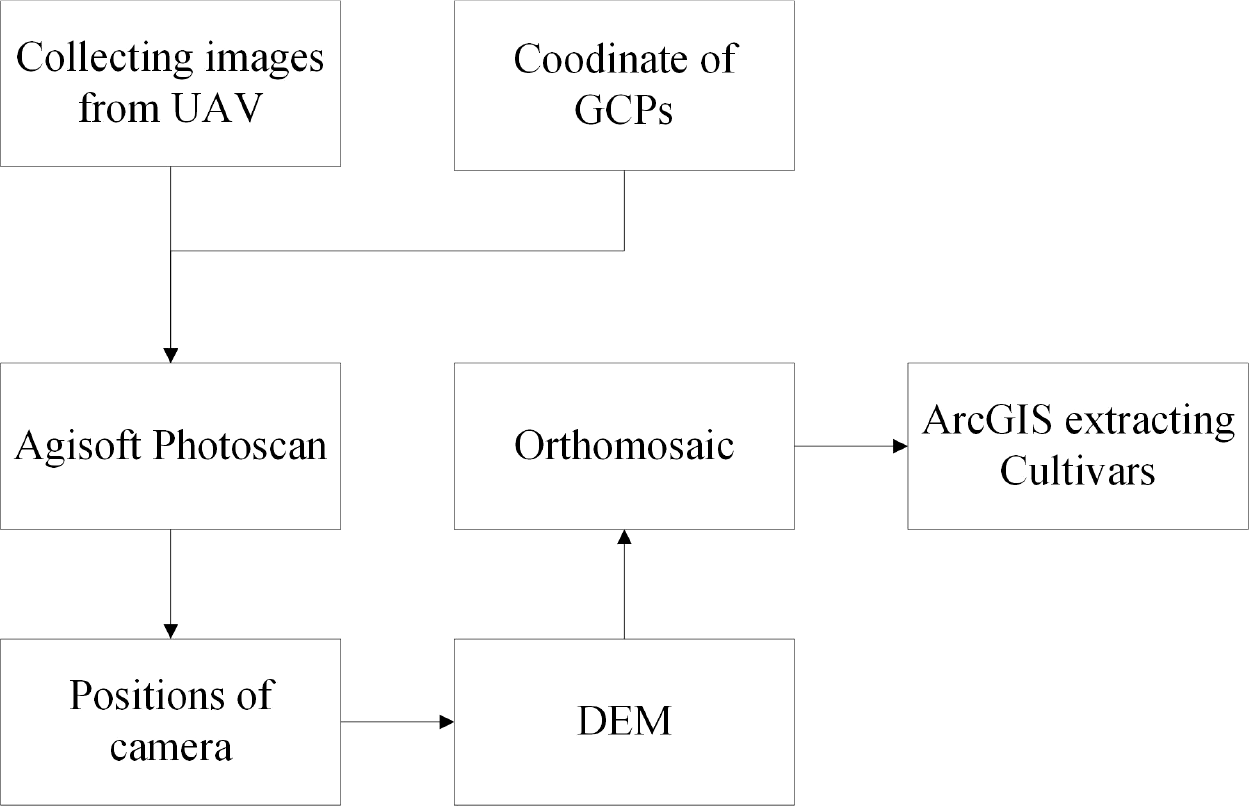
Flowchart of the UAV RGB image mosaic processing.

(i) Stitching images. Agisoft Photo-Scan Professional Version 1.2.2 (Agisoft LLC) was used to stitch images, it can process photos based on multi-view 3D reconstruction technology. (ii) Georeferencing used map coordinates to locate the image. By georeferencing raster data, multi-period raster data can be viewed, queried, and analyzed together. Typically, raster data is georeferenced using existing spatial data in the desired map coordinate system. Images from August 1, 2020 were used in this study. This process involved identifying a series of GCPs (with known x, y coordinates) to link the location of the raster dataset with the location of the spatial reference data (target data). The control points were precisely identifiable locations in the raster dataset and actual coordinates. More than 10 coordinate points for each plot were selected for georeferencing in this study. (iii) The images were cropped after georeferencing using ESRI ArcGIS 10.7 (Redlands, CA, USA) to outline the mask data, the software can be used to create maps, perform spatial analysis, and manage data. Since each plot was not strictly the same size during pre-planting, the specific criteria for outlining plots were to remove roads and furrows. Interactive data language (IDL) was then used to crop the images. The IDL code was written by ENVI 5.3 (Exelis Visual Information Solutions, L3Harris Geospatial, Boulder, CO, USA) (**Figure 4**). ENVI provides professional spectrum analysis tools, extended functions can also be written using IDL.

#### 2.2.4 Vegetation indices Extraction

The vegetation indices were extracted using RGB images and MATLAB R2021a (MathWorks, Portola Valley, CA USA). Vegetation Indices can be constructed to enhance the interpretation of remote sensing images through the linear or nonlinear combination of two or more characteristic bands and play an important role in crop growth monitoring. ExG and ExGR are two common visible bands used as vegetation indices. They provide a near binary intensity image that outlines the vegetation area of interest, and then the vegetation information can be extracted through threshold segmentation. The color index of vegetation extraction (CIVE) integrates the red, green, and blue bands to enhance the vegetation information. In addition, the Green-Red Ratio Index (GRI), Green-Red Vegetation Index (GRVI), Modified Green-Red Ratio Vegetation Index (MGRVI), Visible Atmospheric Resistance Index (VARI), and Warbeck Index (WI) change the linear exponential to form a ratio to enhance the differences among different cultivars. The Soil Adjusted Vegetation Index (SAVI) reduces the sensitivity of soil to traditional vegetation indices and thus, reduces their impact. The red green blue VI (RGBVI) is defined as the normalized difference of the squared green reflectance and the product of blue × red reflectance. The vegetative index (VEG) was designed to manage the variability of natural daylight illumination. The VDVI (visible-band difference vegetation index) was constructed based on the normalized diffuse vegetation index (NDVI) but only used the visible band, which can render the vegetation and non-vegetation index values more compact. comb1 and comb2 are indices that are specifically used to determine greenness. They are linear combinations of ExG, ExGR, CIVE, and VEG. The plant pigment ratio (PPR) is an index that produces an output image in which strongly pigmented foliage presents a high PPR, while weakly pigmented foliage has a low PPR. In summary, these 15 visible bands vegetation indices were used in this study as shown in **Table 2**.

**Table 2:** Summary of the RGB-based vegetation index.

VI	Abbreviation	Equation	Reference
R_n_	R_n_	=R/R(max)	Arroyo, Guijarro, & Pajares et al. (2016) (Arroyo et al., 2016)
G_n_	G_n_	=G/G(max)	
B_n_	B_n_	=B/B(max)	
r	r	=R_n_/(R_n_+G_n_+ B_n_)	Guijarro et al. (2011) (Guijarro et al., 2011)
g	g	=G_n_/(R_n_+G_n_+ B_n_)	
b	b	=B_n_/(R_n_+G_n_+ B_n_)	
Color Index of Vegetation Extraction	CIVE	=18.78745+(0.44r)-(0.88g)+(0.385b)	Kataoka et al. (2003) (Kataoka et al., 2003)
Combined 1	COMB1	=(0.25ExG)+(0.3EXGR)+(0.33CIVE)+(0.12VEG)	Guijarro et al. (2011)
Combined 2	COMB2	=(0.36ExG)+(0.47CIVE)+(0.17VEG)	Guijarro et al. (2011)
Excess Green	ExG	=(2g)-r-b	Woebbecke et al. (1995) (Woebbecke et al., 1995)
Excess Green Minus Excess Red	EXGR	=ExG-(1.4r)-g	Guijarro, & Pajares (2011) (Guijarro et al., 2011)
Ratio Green Red Index	GRI	=G/R	Li et al. (2010) (Li et al., 2010)
Green Red Vegetation Index	GRVI	=(G-R)/G+R	Gitelson et al. (2002) (Gitelson et al., 2002)
Modified Green Red Vegetation Index	MGRVI	=(G^2^-R^2^)/(G^2^+R^2^)	Bendig et al. (2015) (Bendig et al., 2015)
Plant Pigment Tatio	PPRb	=(G-B)/(G+B)	Metternicht et al. (2010) (Metternicht, 2010)
RGB Vegetation Index	RGBVI	=((G^2^-(R*B))/((G^2^+(R*B))	Bendig et al. (2015)
Soil Adjusted Vegetation Index	SAVI	=1.5(G-R)/(G+R+0.5)	Li et al. (2010)
Visible Atmospherically Resistant Index	VARI	=(G-R)/(G+R-B)	Gitelson et al. (2002)
Visible Difference Vegetation Index	VDVI	=(2G-R-B)/(2G+R+B)	Wang et al. (2015) (Wang et al., 2015)
Vegetation Index	VEG	=g/r^a^b^(1-a)^∴a=0.667	Hague, Tillett, & Wheeler (2006) (Hague et al., 2006)
Woebbecke Index	WI	=(g-b)/|r-g|	Woebbecke et al. (1995)

R, G, and B represent the DN variables from digital image red, green, and blue bands, respectively.

#### 2.2.5 Canopy coverage extraction

Threshold segmentation is a simple method to extract interesting regions from grayscale images. In this experiment, the binary images were generated using the excess green index (EGI, (2G-R-B)/G). The threshold value (Green_threshold_) was established as 0.05 for canopy cover and background segmentation. First, the EGI was calculated using the three channels (R, G, B) of the image, and the EGI values > the green threshold corresponded to the canopy (binary value = 1). The values < Green_threshold_ corresponded to the soil (binary value = 0). The percentage of canopy (binary value = 1) pixels in an image was calculated as the canopy coverage. Finally, the canopy coverage of all the plots was calculated in turn.

#### 2.2.6 Crop height extraction

**Figure 5:**
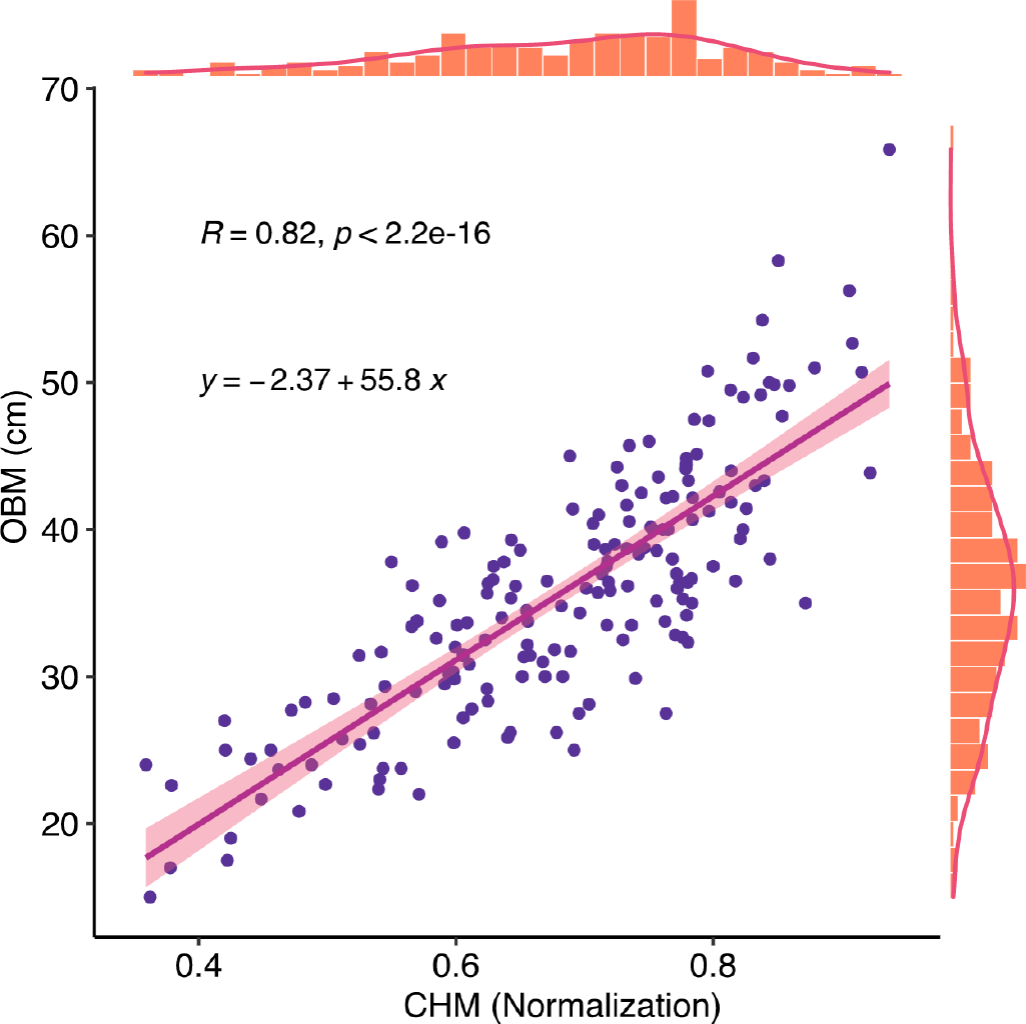
Correlation between CHM and Observation. CHM is crop height model, it is the plant height estimated data.

In this study, crop height (CH) was extracted from the RGB image point clouds and used as a canopy structure indicesto estimate the yield parameters. UAV-based RGB images were collected at an earlier stage of plant emergence on August 1, 2020, to create a bare ground DEM using a photogrammetric three-dimensional (3-D) point cloud. Height peaks within the plot were segmented using the Otsu algorithm (Qiao and Sun, 2014), and the lower peaks were used as the bare ground height. Digital surface models (DSM) were created from UAV-based RGB images collected after August 1, 2020. After that, a crop height model (CHM) was obtained from pixel-level subtraction of the DSM and DEM with subsequent normalization. A total of 190 manually collected ground CH measurements were used to assess the accuracy of the CHM (**Figure 5**). OBM is manually collected crop height. The distribution of CH reflects the different genotypes and the heterogeneity of soybean fields.

#### 2.2.7 Texture information extraction

The texture information was extracted using Python version 3.8 (Python Software Foundation, Wilmington, DE, USA) and PyCharm version 2021.3.3 (JetBrains, Prague, Czechia). The texture is a common method used to extract image information in digital image processing. Although there is no formal definition of texture, this descriptor intuitively measures its properties. Calculating the texture consists of two methods: statistical and spectral methods, with statistical methods producing texture information, such as smoothness, roughness, and graininess. Spectral methods are based on the nature of Fourier spectrum and focus on detecting global periodicity in an image by identifying high-energy narrow peaks in the spectrum (Bharati et al., 2004). The spectral texture information of the canopy was calculated using RGB data. This texture information was extracted from the RGB-based grayscale co-occurrence matrix (GLCM) of the red, green, and blue bands. The GLCM texture consists of nine indices, including the mean, variance, homogeneity, contrast, dissimilarity, entropy, energy, correlation, and autocorrelation. The processing window is 3 rows x 3 columns. To obtain more texture information, the mean, minimum, maximum, SD, and coefficient of variation (CV) values of the GLCM metrics within each plot were calculated. Thus, nine (GLCM indices) × three (bands) × five (statistical metrics) = 135 RGB texture variables that were generated.

#### 2.2.8 Indices selection

Indices selection is an important step in machine learning. The indices determine the upper bound of the model if the algorithm determines the lower bound of it. In addition, it is important to note that the model with as many indices as possible will not always perform better. Sometimes several indices will provide the desired result, and the high correlation indices cannot always be deleted directly to obtain a better performing model. This study tried many methods to filter the indices. Boruta (Kursa and Rudnicki, 2010) used a random forest approach based on the Boruta algorithm to screen the initial 72 Indices, the complete list of indices is given in the supplementary table (**Table S1**). It disrupts the order of indices and calculates the importance of indices. In this study, the lodging and canopy cover were not participants in the screening process. The indices matrix was then shuffled, and the shadow indices were combined with the real indices to form a new Indices matrix. The indices were selected or removed in order of importance.

The steps for running the Boruta algorithm are as follows:

1. First, it adds randomness to a given dataset by creating mixed copies of all the indices (shuffled features).
2. It then trains an extended dataset for random forest classification and uses an indices importance measure (set by default to an average reduction in precision) to evaluate the importance of each indicator, with a higher meaning indicating greater importance.
3. In each iteration, it checks whether a true indices has higher importance than the best-shaded indices (whether the indices scores higher than the largest shuffled indices) and keeps removing indices that it considers to be very unimportant.
4. Finally, the algorithm stops when all the indices have been confirmed or rejected, or when the algorithm reaches a prescribed limit for the operation of the random forest.

Boruta follows all the relevant indices selection methods, which capture all the indices related to the outcome variable. In contrast, most traditional indices selection algorithms follow a minimal optimization approach, which relies on a small subset of indices and produces a minimal error in selecting the classification. Compared to traditional indices selection algorithms (recursive feature elimination algorithms [RFE] (Liu and Wang, 2021)). Boruta can generate better results on the importance of the indices and is also easy to interpret.

#### 2.2.9 Data filtering

Outliers from the remaining indices were removed using the One-Class-SVM outlier detection algorithm, which uses the appropriate Python version 3.8 (Python Software Foundation, Wilmington, DE, USA) packages and environment. One-class-SVM uses a spherical rather than a planar approach and the algorithm obtains a spherical boundary around the data in the feature space, a hypersphere whose volume is minimized, thus minimizing the effect of outliers.

One-Class-SVM (Tax and Duin, 1999) can be a good outlier detection method when the data dimensionality is very high, or there are no assumptions about the distribution of the data. It finds the hyperplane for the partition and the support vector machines using the support vector domain description (SVDD) idea. All the samples that are not anomalies are expected to be positive classes for SVDD, and it uses a hypersphere instead of a hyperplane to divide. The algorithm obtains a spherical boundary around the data in the feature space and expects to minimize the volume of this hypersphere, thus, minimizing the effect of outlier data. It is possible to determine whether the data is within or not after the LaGrange dual solution was used. If the distance ≤ to the radius, it is not an anomaly, and if it is outside the hypersphere, it is an anomaly.

In this study, a radial basis function kernel is used with a gamma value of 0.001, a lower bound on the support vector fraction of 0.03, a residual convergence condition of 1e^-3^, and the remaining parameters as default. The values are based on experience or multiple attempts to select the most suitable parameter value. To satisfy this condition means wrapping all the suitable data points in the sphere to achieve unsupervised outlier detection.

In scikit-learn version 0.23.2 (Pedregosa et al., 2012), One-Class-SVM (Pedregosa et al., 2012) was used to detect outliers using the SVM package.

#### 2.2.10 Machine learning

Five machine learning algorithms were used to estimate yields in this study, including support vector machine regression (SVM), logistic regression (Logistic), random forest regression (RFR), partial least squares regression (PLSR), and DNN networks. All the model methods are based on the R language version 4.1.1 (R Foundation, Vienna, Austria) with the computation of DNN network using the h2o package version 3.34.0.3 (H2O.ai, Mountain View, CA, USA) package, which is based on Java but provides a computational interface between the R and Python. A total of 1,615 cultivars were divided into two parts, 0.7 and 0.3, as the training set and the validation datasets.

In this study, there were five principal components before calculations to elucidate the mean center of all the data in PLSR. During the process of data processing, leave-one-out cross-validation was performed serially; the cross-validation was optimized for speed, and some generality was sacrificed. In particular, the model matrix was calculated only once for the complete cross-validation. The jackknife method was also used to correct for bias (Seasholtz and Kowalski, 1992).

The Logistic were 10 folds. The first was to obtain the lambda sequence and calculate the result of the fit omitting each fold. The errors accumulated, and the mean error and SD of the folds were calculated. The alignment is “lambda”, and the lambda values from the autonomous fit (all data) were used to rank the predicted values from each fold (Simon et al., 2011). The other parameters were set to default.

The random forest is the randomization of column variables and row observations of a dataset to generate multiple classifiers, and finally, the classification tree results are aggregated. Two important parameters are the number of variables preselected by tree nodes and the number of trees in the random forest. In this study, the number of variables preselected by the tree nodes was one-third of the dataset, and there were 500 trees in the forest (Edwards et al., 2018).

SVM can be formalized as a problem of solving convex quadratic programming and is also equivalent to the problem of minimizing the loss function of a regularized hinge. The learning algorithm of SVM is the optimization algorithm for solving convex quadratic programming. Radial was used as the kernel function here; gamma is 1/(data dimension); the termination criterion was 0.001, and epsilon in the insensitive-loss function was 0.01 (Fan et al., 2005).

DNN is a feedforward multilayer artificial neural network. In this study, the hidden layer has two hidden layers, each with 200 neurons, and 10 iterations are needed to make the network converge; the target ratio of communication overhead to computation was 0.05, and the learning rate was 0.005. These parameter settings are based on empirical or model-recommended optimal parameters (Xu et al., 2016).

#### 2.2.11 Statistical indices

The coefficient of determination (R^2^), root mean square error (RMSE) and relative root mean square error (rRMSE) were selected to test the training and validation models (Liu et al., 2021).

## 3 Results

### 3.1 Selection of the best estimation dates

**Figure 6:**
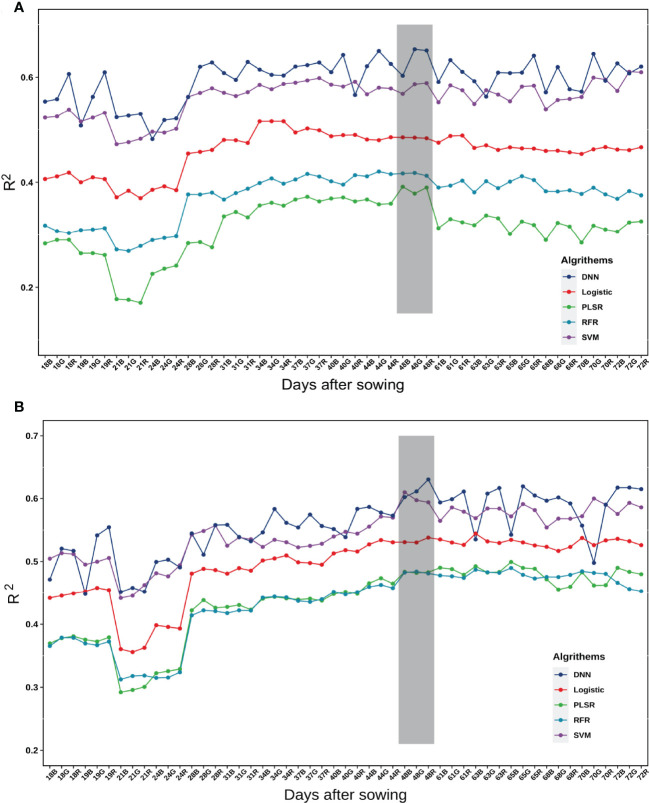
Accuracy of the estimation of different models on yield parameters at different dates. **(A)** Grain number of seeds per plant. **(B)** Grain weight per plant. The digital numbers represent the days after sowing, and R, G, and B represent the spectral bands. The grey background represents the selected optimal estimation model from day 48.

All the indices (162 for every date) were used to identify the most suitable time point to estimate the yield parameters. Data were collected on 17 dates. Each of the data points for 17 days was used to provide a result to estimate the yield parameters. In general, the estimations for grain number per plant were poor before emergence to second trifoliolate stage (21 days after sowing), with a gradual increase at the third trifoliolate to the sixth trifoliolate stage (24~44 days after sowing) until the peak at flowering stage (48 days), and then a slow decline. The trend of the grain weight per plant was the same before flowering stage (44 days after sowing). The changes after seeds to maturity stage (48 days after sowing) were not obvious, and there were many abrupt changes. This was probably owing to the changes in the pixel colors that were impacted by weather. In addition, they can be affected by maturity (**Figure 6**). In this study, we wanted to obtain the earliest time point as the result, so on balance we chose the time point with the highest accuracy for early estimation and the critical fertility period of common interest in soybean production as the most appropriate time point for estimating yield parameters.

### 3.2 Selection of indices

The 48-day red band texture information and RGB vegetation indices were selected. These 72 indices (**Table S1**) included 45 texture indices, 24 RGB vegetation indices, canopy cover, crop height, and lodging.

**Figure 7:**
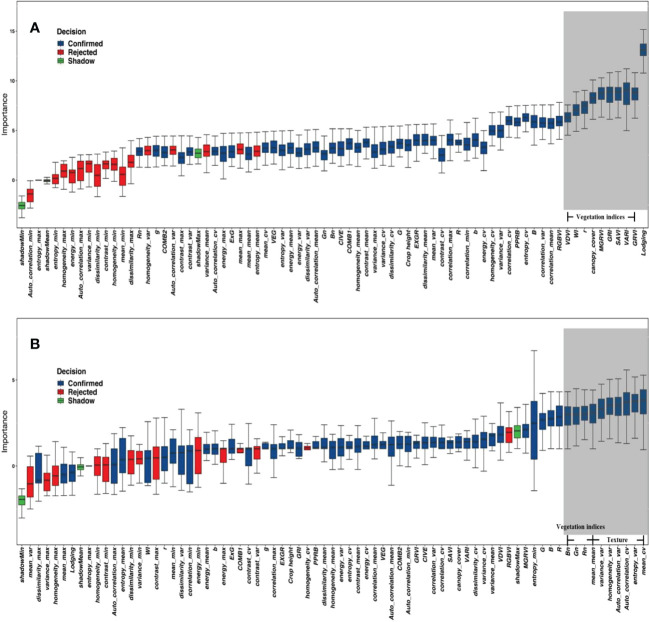
The importance of indices for yield parameters. **(A)** Grain weight per plant. **(B)** Grain number of seeds per plant. The red box plot was the indices recommended for removal; the blue box plot was the Indices recommended to be retained. The grey background represents the top 10 indices.

The indices of grain number of seeds per plant and the grain weight per plant were screened separately using the Boruta algorithm, which gave an average ranking of importance and a recommendation to eliminate data points (red) or retain them (blue) based on the results of 100 times ranking. The top indices for the grain weight per plant were the texture indices, while the vegetation indices were in the middle. Out of the 100 times importance rankings provided by the Boruta algorithm, indices with 80 times importance rankings below the shadow indices were marked red and removed. Thus, the nine indices associated with the maximum and minimum values in the red box plot in **Figure 7B** were deleted. The top importance for the grain number of seeds per plant was vegetation indices. The top one was lodging, and crop height was also advanced, but all the texture indices were ranked at the bottom (outside the top 10). The analysis of the grain weight per plant was the same as the grain number of seeds per plant. The nine indices that were deleted were the texture indices related to the maximum and minimum values with the red box plot in **Figure 7A**.

### 3.3 Study on the estimation of yield parameters by different indices and machine learning algorithms

#### 3.3.1 Grain number of seeds per plant

The spectral information extracted from the RGB images was used to obtain texture information, canopy structure information, and crop height. Machine learning algorithms (support vector machines, random forests, partial least squares, logistic regression, and DNN neural networks) were used to estimate the grain number of seeds per plant in soybean.

As shown in **Table 3**, the combination of spectral and canopy structure information improved the accuracy of estimation of yield parameters. As the number of indices increased, the R^2^ increased, resulting in a decrease in both the RMSE and rRMSE. The accuracy of estimation improved with the addition of texture indices for support vector machines and the DNN, while the others had limited changes. All the models had roughly the same optimization with the addition of crop height, and a significant change with the addition of lodging.

**Table 3:** The accuracy of estimation of different models for the grain number of seeds per plant.

Type	No. of indices	Metrics	PLSR	RFR	Logistic	SVM	DNN
VIs	25	R^2^	0.40	0.37	0.41	0.44	0.43
		RMSE	26.69	27.46	26.41	25.98	26.30
		rRMSE	45.00%	46.30%	44.60%	43.92%	43.84%
VIs+TI	60	R^2^	0.41	0.37	0.45	0.55	0.58
		RMSE	26.38	27.31	25.55	23.41	24.27
		rRMSE	44.52%	46.13%	43.15%	39.56%	42.34%
VIs+TI+CC	61	R^2^	0.42	0.37	0.45	0.55	0.58
		RMSE	26.36	27.37	25.52	23.41	22.51
		rRMSE	44.56%	46.25%	43.17%	39.58%	38.76%
VIs+TI+CC+CHM	62	R^2^	0.43	0.39	0.46	0.56	0.61
		RMSE	25.97	26.98	25.16	23.11	21.50
		rRMSE	43.80%	45.50%	42.50%	39.00%	38.00%
VIs+TP+CC+CHM+Lodging	63	R^2^	**0.45**	**0.41**	**0.48**	**0.58**	**0.66**
		RMSE	**25.39**	**26.39**	**24.76**	**22.5**	**21.00**
		rRMSE	**42.97%**	**44.65%**	**41.86%**	**41.80%**	**41.70%**

VIs, vegetation index; TI, texture information; CC, canopy cover; CHM, crop height; DNN, deep learning network; Logistic, logistic regression; PLSR, partial least square regression; RFR, random forest regression; SVM, support vector machine regression. The best results in terms of R^2^, RMSE, and rRMSE values through different indices with various modeling methods are shown in bold.

All the model methods performed similarly for single categories of indices. The SVM method performed slightly better, but the DNN network quickly outperformed the SVM when the indices were increased. They were clearly split into three levels. The RFR and PLSR methods were relatively poor, and the logistic method was in the middle, while the SVM and DNN methods performed better. The best yield estimates were r^2^ = 0.66 and rRMSE=32.62% when the DNN method was used with 63 Indices (**Figure 11**).

The r^2^ gradually increased as the number of input indices increased, and the rRMSE gradually decreased for all the regression methods, indicating that all the methods can handle the fusion of multimodal data to some extent. The DNN outperformed other methods because of the large amount of data in this study, but it has been demonstrated (Bonazza et al., 2019) that traditional machine learning algorithms may work better when there is less data. The DNN tends to perform better when analyzing larger sample sizes and more complex non-linear datasets.

**Figure 8:**
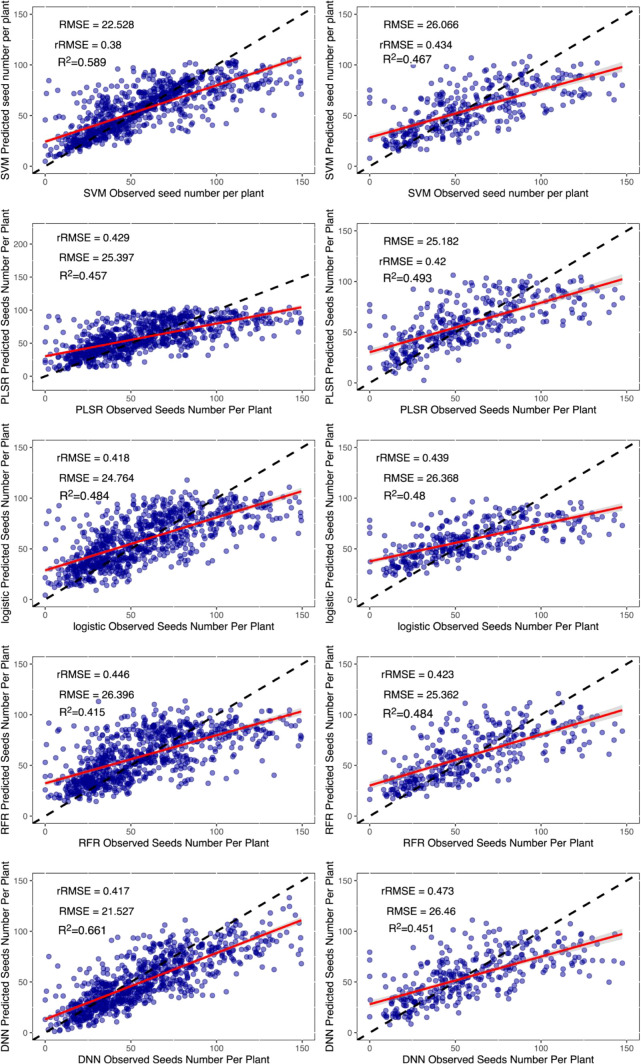
Results of machine learning algorithms to estimate the grain number of seeds per plant. The left column is the training dataset, and the right column is the validation dataset. The black dashed line is the 1:1 line, and the red solid line is the data fitted line. SVM, support vector machines regression; RFR, random forests regression; PLSR, partial least squares regression; Logistic, logistic regression; DNN, deep neural network.

Although the DNN neural network also performed well at estimating the grain number of seeds per plant, there was a decrease in the validation dataset, which could be owing to errors in the manual measurement of data (**Figure 8**).

A comparison of the estimated dataset from each regression method with the observed dataset indicated that each method had a similar distribution of estimated yield parameters. In common with the performance of the existing studies (Maimaitijiang et al., 2017), all the regression methods underestimated the higher yield parameters of cultivars. Theoretically, this should not be an issue because we used flowering data in which the nutritional growth had ceased. In theory, there was no longer a significant increase in pod number. However, 48 days may not be the flowering period for all the cultivars, and some flowered late. This resulted in the lack of determination of the grain number of seeds per plant during this period (**Figure 8**).

#### 3.3.2 Grain weight per plant

As shown in **Table 4**, the combination of all the information (r^2^ = 0.64 in the training dataset and r^2^ = 0.49 in the validation dataset) resulted in the most accurate estimation, and the accuracy of the model increased with each additional category of indices. Moreover, unlike the grain number of seeds per plant, the 48-day vigorous growth state (specifically the photosynthetic state) determined the final amount of biomass that accumulated in the final plant to some extent, i.e., the result of the grain weight per plant.

**Table 4:** The accuracy of estimation of different models for grain weight per plant.

Type	No. of indices	Metrics	PLSR	RFR	Logistic	SVM	DNN
VIs	25	R^2^	0.43	0.42	0.45	0.48	0.48
		RMSE	3.98	3.97	3.98	3.83	3.88
		rRMSE	42.96%	42.53%	41.42%	41.26%	43.52%
VIs+TI	60	R^2^	0.45	0.45	0.51	0.58	0.58
		RMSE	3.89	3.86	3.67	3.4	3.541
		rRMSE	41.72%	41.31%	39.33%	36.34%	39.45%
VIs+TI+CC	61	R^2^	0.45	0.46	0.51	0.59	0.61
		RMSE	3.9	3.85	3.67	3.40	3.28
		rRMSE	41.75%	41.26%	39.32%	36.47%	35.29%
VIs+TI+CC+CHM	62	R^2^	0.48	0.49	0.52	0.61	0.62
		RMSE	3.78	3.74	3.60	3.31	3.25
		rRMSE	40.4%	40.00%	38.52%	35.40%	35.32%
VIs+TI+CC+CHM+Lodging	63	**R^2^**	**0.48**	**0.49**	**0.53**	**0.62**	**0.64**
		**RMSE**	**3.78**	**3.76**	**3.6**	**3.287**	**3.16**
		**rRMSE**	**40.43%**	**40.22%**	**38.54%**	**35.18%**	**33.80%**

VIs, vegetation index; TI, texture information; CC, canopy cover; CHM, crop height; DNN, deep neural network; Logistic, logistic regression; PLSR, partial least squares regression; RFR, random forest regression; SVM, support vector machine regression. The best results in terms of R^2^, RMSE, and rRMSE values through different sensors with various modeling methods are shown in bold.

**Figure 9:**
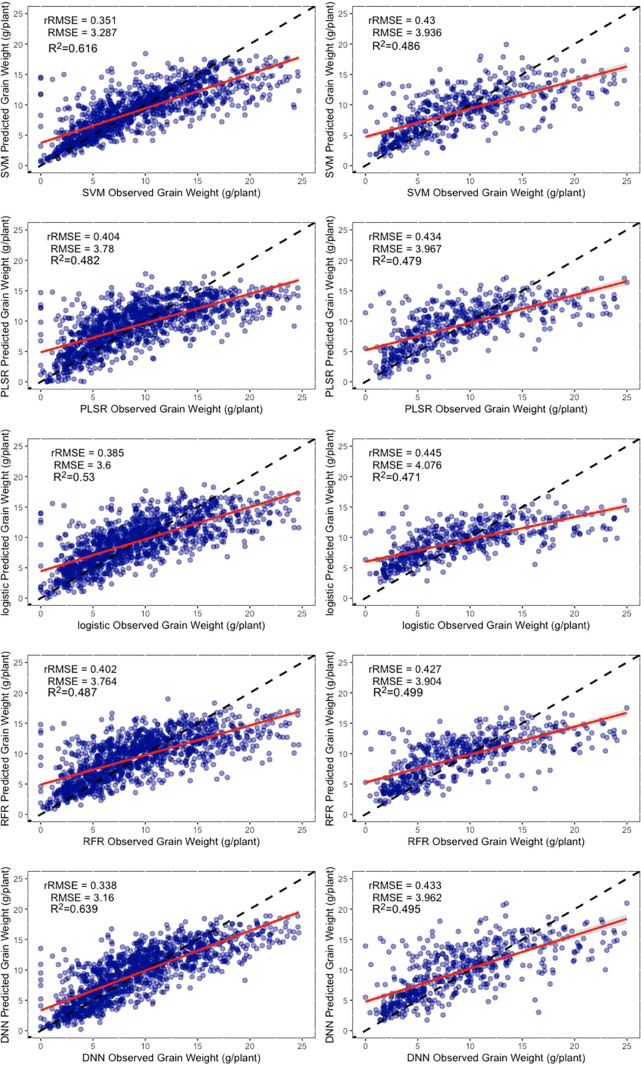
Results of the machine learning algorithms to estimate the grain weight per plant. The left column is the training dataset, and the right column is the validation dataset. The black dashed line is the 1:1 line, and the solid red line is the data fitted line. SVM, support vector machines regression; RFR, random forest regression; PLSR, partial least squares regression; Logistic, logistic regression; DNN, deep neural network.

The combination of all the information was more accurate than single category data for estimating the yield regardless of the method used. The r^2^ ranged from 0.47 to 0.63 where the grain weight per plant was more accurate than the grain number of seeds per plant, possibly because the plant nutrition influenced by canopy information was more likely to change the grain weight than the grain number of seeds. However, the texture information had a more pronounced effect on the grain weight than the grain number of seeds, possibly because the texture is related to photosynthesis (Heckmann et al., 2017). It should be noted that the improvement in the estimation of yield parameters with texture information compared with the vegetation indices was not only substantial but suggested that, although some methods have been used to remove redundant data, it still contains a large proportion of redundant information (**Figure 9**).

**Figure 10:**
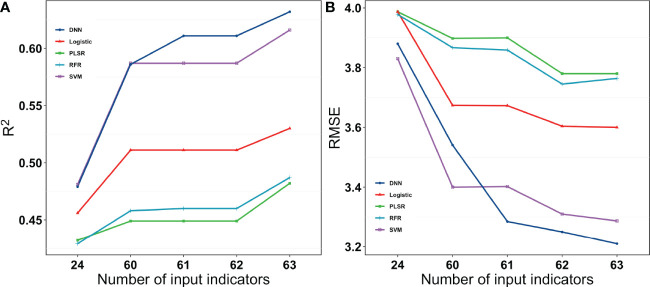
R^2^ and RMSE for the different models with different input indices for grain weight per plant. 24 were vegetation indices. 60 were vegetation indices and texture indices. 61 were vegetation indices, texture indices, and canopy cover. 62 were vegetation indices, texture indices, canopy cover, and crop height. 63 were vegetation indices, texture indices, canopy cove, crop height, and lodging.

When all the data were combined to estimate yield, the DNN neural network performed better for both the grain number of seeds per plant and the grain weight per plant. DNN outperformed the other machine learning algorithms for both training datasets and validation datasets by a large margin. SVM also performed well on the training dataset, but it performed the validation dataset less accurately (**Figure 10**).

Unlike the grain number of seeds per plant, the estimation for grain weight was not underestimated at higher values. There are two likely reasons for this. (1) They were easier to measure and gave more accurate results without too many outliers. (2) The state of canopy was a greater determinant for the formation of grain weight. There could be a deeper reason for this different performance in estimating the grain number of seeds per plant and grain weight per plant, and more experiments can be designed to determine the answer.

## 4 Discussion

The optimal time point to estimate the yield parameters in this paper was 48 days (flowering). In maize and rice studies, Li et al. (2021) showed that crop yields can be satisfactorily forecasted one to three months before harvest (Li et al., 2021). Zhou et al. (2017) used three soybean cultivars to produce results that indicated that the best estimation period is the gestation period (Zhou et al., 2017). Previous research has provided results comparable to those of this study. However, there are some differences between them that could be attributed to two reasons. (1) The soybeans are dried by hand after harvest, which usually takes place when the plants are still green. Therefore, it is difficult to obtain data for soybeans during this stage. (2) In earlier studies, the limitation of the number of cultivars (usually < 5) resulted in the spectral information of the flowering period not being very distinguishable, while the later stage effects on yield were more obvious owing to the cumulative effects. That suggests that the results of these studies could be limited. In contrast, this study found that the flowering period was the best period to distinguish the cultivars, and the spectral information was more easily distinguished in this stage. Physiologically, this result is also supported by the fact that the flowering stage is a peak period for soybean growth and development. Although 48 days after sowing was used as the base data in this paper, in the future, a combination of Indices with a high correlation of yield (possibly from different times) could improve the accuracy of estimation. However, this would increase the difficulty of interpreting the established models to estimate yield. Another option could be to use a more reasonable time series-based model approach.

Previous studies used correlation (Hall, 1999) or recursive feature elimination (RFE) (Granitto et al., 2006) algorithms to remove indices. In comparison, the Boruta algorithm removed Indices that were maximum or minimum values for texture information. These values are usually unchanged or changed only slightly. They provide almost no information and create considerable interference information in the estimation results, thus, affecting the accuracy of estimating the experimental results. Overall, seven of the top 10 indices of the importance for grain weight per plant were texture indices, suggesting that the canopy structure is more influential for grain weight per plant than the vegetation indices. The mean of texture (top one) information reflects the degree of the neatness of growth, and a neater state of growth is likely to result in a higher grain weight per plant. The top 10 importance indices for the grain number of seeds per plant were vegetation indices that reflected the physiological status, while all the texture indices ranked lower, with lodging ranked first, followed closely by GRVI and VARI, which are all closely related to the biomass or NDVI. Zhou et al. (2017) also found that NDVI-related indices had a higher effect on yield (Zhou et al., 2017), and this study found that this effect could be attributed more to the grain number of seeds per plant than the grain weight per plant. Canopy cover reflects the ability of the vegetation to receive energy, suggesting that the state of the soybean itself may have a more pronounced effect on the grain number of seeds per plant. The effect of lodging on the grain number of seeds per plant was much greater than that for grain weight per plant, indicating its importance. The effect of lodging on yield has been evaluated very ambiguously in traditional agronomic research. Cooper (1971) studied the effect of early lodging on yield outcomes and identified a 21% decrease in yield (Cooper, 1971). However, they did not address the effect of lodging in more detail. This is primarily because lodging is a combination of several complex traits. A separate discussion of the different parameters of yield data could be a valid approach, and existing evaluation indices should be updated or reconstructed.

**Figure 11:**
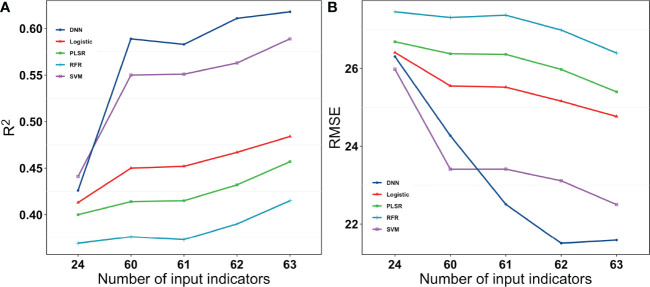
R^2^ and RMSE for different models with different numbers of input Indices for the grain number of seeds per plant. 24 were vegetation indices. 60 were vegetation indices and texture indices. 61 were vegetation indices, texture indices, and canopy cover. 62 were vegetation indices, texture indices, canopy cover, and crop height. 63 were vegetation indices, texture indices, canopy cove, crop height, and lodging.

In contrast to the previous study, Maimaitijiang et al. (2020) also used some algorithms. They found less of a difference between the different algorithms, with an R^2^ that ranged from 0.65 to 0.72 (Maimaitijiang et al., 2020). This could be because their results were based on only three cultivars. However, this study was based on a large number of cultivars to better distinguish the performance of the different algorithmic models. As for **Figures 10** and **11**, the five machine learning algorithms were clearly divided into three parts, which exhibit very different results. In addition, machine learning methods performed similarly to DNN-based regression when fewer indices were inputted, while the DNN outperformed other methods when more indices were utilized. An increase in the number of indices resulted in an increase in R^2^ and a decrease in RMSE for all the regression methods. However, the magnitude of increase or decrease varied. The DNNs performed better than the other regression algorithms owing to the large amount of data in this study, but they do not have a very clear advantage when the dataset is smaller. This could be owing to the ability of deep learning to often outperform machine learning methods when processing larger sample sizes and complex non-linear datasets.

This work has some practical implications, firstly it given more accurate conclusions of existing modeling methods based on >1,500 cultivars, and we found a pronounced stratification of the model, which will have a positive impact between the technology and practical production. Secondly, instead of estimating traditional yields, we estimated two yield parameters and found that the effect of lodging was different, it provided with ideas for doing some other complex traits later. Finally, we evaluated the accuracy of the estimated yield parameters with early stage, these allowing breeders to evaluate the performance of specific cultivars at an earlier stage.

Although this study has extracted as many indices as possible to estimate the final yield parameters, the RGB itself contains limited information, which resulted in the poor accuracy of estimation. In addition, the diversity of cultivars that numbered >1,500 exacerbated this result. But RGB is more economical and practical than multiple spectral sensors, and it is impractical to use hyperspectral in large test field currently. Although existed hyperspectral methods will get better results, they are often obtained by only 5-10 cultivars and have not been validated over a large number of cultivars. Although only RGB data were used in this study, our conclusions will be more realistic and credible based on data from more than 1500 cultivars, apart from this, the process of moving from method to field may require more experiments, such as multi-year and multi-point experiments. In this study, the grain number of seeds per plant and the grain weight per plant were used as yield parameters instead of the traditional plot yield. There were several reasons for this. First, analyzing such a large number of cultivars, including the control cultivars, that totaled 1,805 plots, with the land arrangement and post-survey was an enormous task. The plot size could only be reduced to one plot with two rows. Secondly, the plot yield can be too severely affected by chance factors, including occasional biotic and abiotic effects, such as seedling deficiency and diseases. These factors could have too much influence on the final plot yield, which interfered with the representation of the real yield data. The mean value of the plots was also calculated for different vegetation indices and textures, so that more reasonable yield parameters were used as the final indicator. The grain number of seeds per plant and grain weight per plant would also be easier to interpret. Use of the Boruta algorithm to eliminate indices is not the best way to do this, but it will provide suggestions for indices in the middle of the range of importance, and the final selection can be artificially conducted. Although the DNN had the best training and accuracy of estimation, the recent rapid development of deep learning algorithms will increase the likelihood of improvements in modeling algorithms. Promising algorithms include the use of long and short-term memory learning (LTSM) or an improved RNN algorithm in combination with time-series data.

## 5 Conclusions

This study explored the potential of RGB data to estimate yield parameters. UAVs provide canopy spectra, structure, and texture information, and five machine learning methods were used to estimate the yield parameters under lodging conditions. The main conclusions are as follows:

1. The most accurate time point for yield parameters estimation is 48 days after sowing when most cultivars are flowering. However, not all the indices correlate best with yield parameters at the flowering stage.
2. A combination of all the screened indices most effectively estimated the yield parameters. Spectral information offers a substantial potential for estimating the yield parameters, and the accuracy of estimating higher yield parameters is the highest when all the information indices are used.
3. The DNN-based model outperforms the PLSR, RFR, logistic, and SVM when the input indices are increased.
4. The effect of lodging levels on yield parameters are significant, and they affect both the grain number of seeds per plant and the grain weight per plant. However, the grain number of seeds per plant is more effective at generating accurate results.

The results suggest that there is substantial potential to estimate the yield parameters using multiple types of data fusion combined with deep neural networks. However, there are some limitations. First, as for breeders and cultivators, multi-year multi-area performance is an important indices of model stability, and it is difficult to judge the effectiveness of the model when we have only one year data. Secondly, our model only considered data from one time-point but considering multiple periods data may increase the estimation accuracy of yield parameters. Finally, we were numerically unable to the effect of lodging on yield parameters, so it was impossible to estimate the impact of lodging. To further improve the accuracy of estimating yield parameters, a larger amount of data is needed to support the estimation of yield parameters for so many genotypes. A future goal is to try to use more efficient methods to improve the stability of the estimated yield model.

## Data availability statement

The raw data supporting the conclusions of this article will be made available by the authors, without undue reservation.

## Author contributions

XJ, LQ, YL, DB, and DL conceived the research. DB completed the first manuscript version, XJ, YL, and DL contributed most of the key changes to the manuscript text. DB, DL, ZW, MS, BG, YL, QW, SG, and JL made significant contributions to the UAV data collection, data processing, and interpretation of the experimental data. DB, BG and DL led the trial design, management of experimental cultivation and collection of agronomic data at harvest. All authors contributed to the article and approved the submitted version.
