# Supplementary material for: Estimation of soybean yield parameters under lodging conditions using RGB information from unmanned aerial vehicles
Source: Front Plant Sci. 2022 Dec 13;13:1012293. doi: 10.3389/fpls.2022.1012293 (PMC9795850; doi:10.3389/fpls.2022.1012293)
Supplement: Supplementary file 1 [file Table_1.docx]

Supplementary Material

# Supplementary Tables

Table S1 Details of 72 indicators

| Type | Category | Name |
| --- | --- | --- |
| RGB VIs | R_n_ | R_n_ |
|  | G_n_ | G_n_ |
|  | B_n_ | B_n_ |
|  | r | r |
|  | g | g |
|  | b | b |
|  | CIVE | Color Index of Vegetation Extraction |
|  | COMB1 | Combined 1 |
|  | COMB2 | Combined 2 |
|  | ExG | Excess Green |
|  | EXGR | Excess Green Minus Excess Red |
|  | GRI | Ratio Green Red Index |
|  | GRVI | Green Red Vegetation Index |
|  | MGRVI | Modified Green Red Vegetation Index |
|  | PPRb | Plant Pigment Tatio |
|  | RGBVI | RGB Vegetation Index |
|  | SAVI | Soil Adjusted Vegetation Index |
|  | VARI | Visible Atmospherically Resistant Index |
|  | VDVI | Visible Difference Vegetation Index |
|  | VEG | Vegetation Index |
|  | WI | Woebbecke Index |
| Texture | mean | mean |
|  |  | minimum |
|  |  | maximum |
|  |  | SD |
|  |  | CV |
|  | variance | mean |
|  |  | minimum |
|  |  | maximum |
|  |  | SD |
|  |  | CV |
|  | homogeneity | mean |
|  |  | minimum |
|  |  | maximum |
|  |  | SD |
|  |  | CV |
|  | contrast | mean |
|  |  | minimum |
|  |  | maximum |
|  |  | SD |
|  |  | CV |
|  | dissimilarity | mean |
|  |  | minimum |
|  |  | maximum |
|  |  | SD |
|  |  | CV |
|  | entropy | mean |
|  |  | minimum |
|  |  | maximum |
|  |  | SD |
|  |  | CV |
|  | energy | mean |
|  |  | minimum |
|  |  | maximum |
|  |  | SD |
|  |  | CV |
|  | correlation | mean |
|  |  | minimum |
|  |  | maximum |
|  |  | SD |
|  |  | CV |
|  | autocorrelation | mean |
|  |  | minimum |
|  |  | maximum |
|  |  | SD |
|  |  | CV |
| Others | canopy coverage | - |
|  | crop height | - |
